# Evaluation of *MC1R* high-throughput nucleotide sequencing data generated by the 1000 Genomes Project

**DOI:** 10.1590/1678-4685-GMB-2016-0180

**Published:** 2017-05-08

**Authors:** Leonardo Arduino Marano, Letícia Marcorin, Erick da Cruz Castelli, Celso Teixeira Mendes-Junior

**Affiliations:** 1 Departamento de Genética, Faculdade de Medicina de Ribeirão Preto, Universidade de São Paulo, Ribeirão Preto, SP, Brazil; 2 Departamento de Química, Laboratório de Pesquisas Forenses e Genômicas, Faculdade de Filosofia, Ciências e Letras de Ribeirão Preto, Universidade de São Paulo, Ribeirão Preto, SP, Brazil; 3 Departamento de Patologia, Faculdade de Medicina de Botucatu, Universidade Estadual Paulista “Júlio de Mesquita Filho,”(UNESP) Botucatu, SP, Brazil

**Keywords:** Melanocortin-1 Receptor, The 1000 Genomes Project, next-generation sequencing

## Abstract

The advent of next-generation sequencing allows simultaneous processing of several genomic regions/individuals, increasing the availability and accuracy of whole-genome data. However, these new approaches may present some errors and bias due to alignment, genotype calling, and imputation methods. Despite these flaws, data obtained by next-generation sequencing can be valuable for population and evolutionary studies of specific genes, such as genes related to how pigmentation evolved among populations, one of the main topics in human evolutionary biology. Melanocortin-1 receptor (*MC1R*) is one of the most studied genes involved in pigmentation variation. As *MC1R* has already been suggested to affect melanogenesis and increase risk of developing melanoma, it constitutes one of the best models to understand how natural selection acts on pigmentation. Here we employed a locally developed pipeline to obtain genotype and haplotype data for *MC1R* from the raw sequencing data provided by the 1000 Genomes FTP site*.* We also compared such genotype data to Phase 3 VCF to evaluate its quality and discover any polymorphic sites that may have been overlooked. In conclusion, either the VCF file or one of the presently described pipelines could be used to obtain reliable and accurate genotype calling from the 1000 Genomes Phase 3 data.

## Introduction

The advent of Sanger sequencing in 1977 and its capillary electrophoresis automation provided complete genome sequencing of various species. However, this technique offers limitations: it is expensive and time consuming, and it can only sequence a small number of samples/fragments at a time). Consequently, some difficulties may arise when the goal is to sequence complete genomes for multiple samples, especially in the case of population studies.

The rise of next-generation sequencing (NGS) methods has allowed simultaneous processing of several individuals/regions with high accuracy: sequencing of the same region several times is possible, and the development of various parameters has enabled quality assessment of the generated sequences (Nielsen *et al.*, 2011). This has led to the proposal of many methods, such as emulsion PCR (*e.g.*, 454 – Roche, Ion Torrent – Life) and solid-phase amplification (*e.g.*, Solexa – Illumina) (Metzker, 2010; Vigliar *et al.*, 2015). Decline in sequencing costs has prompted numerous collaborative projects for whole-genome sequencing like the 1000 Genomes Project (The 1000 Genomes Project Consortium, 2012). Such projects use distinct population samples, thereby allowing the discovery of new variants and elucidation of important evolutionary and demographic details that shaped the human genome (Matullo *et al.*, 2013).

Phase 1 of the 1000 Genomes Project, which happened from 2008 to 2010, included a series of research centers that worked together to sequence 1,092 complete genomes from 14 populations. The high cost and genome size meant that these first results consisted mainly of low-coverage whole-genome sequencing, high-coverage exome sequencing, and high-density SNP Array panels. Data treatment and analysis helped to place each genotype into biallelic categories for each sample and each site. By means of an imputation process, linkage disequilibrium analysis of the data aided confirmation/inference or even correction of the variants obtained from low-coverage non-exome data. The retrieved data thus present flaws and bias, such as the lack of some polymorphisms due to limitations of the genotype calling methods available at the time. In addition, the method used to infer genotypes and haplotypes did not enable differentiation of triallelic SNPs and considered them to be biallelic, which decreased the informativity of various *loci* (Castelli *et al.*, 2014). The last phase (Phase 3), released in October 2014, encompassed 2,504 complete genomes and provided multiallelic data for the first time (Sudmant *et al.*, 2015; The 1000 Genomes Project Consortium, 2015). Because many rare variants located within known linkage disequilibrium blocks exist (Howie *et al.*, 2011), studies like the 1000 Genomes Project can serve as basis to infer undetected variants. Low-frequency variants may have 60-90% precision on imputation, even in admixed populations (The 1000 Genomes Project Consortium, 2012). Despite the many improvements achieved on mapping, imputation, and genotype calling, the current NGS technologies still have error rates of, at least, 10^-4^ per nucleotide, which culminates in high rate of false positives (around 5%) when low-coverage sequencing is employed (Matullo *et al.*, 2013).

Several studies have addressed this data analysis issue by using *in silico* methods to identify and correct data for any bias, which may result in mismapping and/or genotype calling errors (Castelli *et al.*, 2014; Gilissen *et al.*, 2012; Nagasaki *et al.*, 2013). In addition, these studies have highlighted the importance of validating the obtained results by using other established and validated methodologies; *e.g.*, Sanger sequencing, for regions of interest. Researchers have developed new scripts and pipelines to detect genotype and haplotype from raw data of the 1000 Genomes Project accurately, which also enabled retrieval of unreleased data from variants overlooked by the Consortium. Different *HLA-G* gene analyses concerning Phase 1 data have validated such scripts, demonstrating higher accuracy of genotype calling in studies of individual genes or delimited chromosome regions (Castelli *et al.*, 2014). Genotype calling accuracy of Phase 3 data has not been approached yet.

Despite the existing errors, data obtained by next-generation sequencing during the 1000 Genomes Project can be valuable for population and evolutionary studies of specific genes. However, the 1000 Genomes dataset is not suitable for the identification of mosaicism or somatic mutations that could be involved in diseases such as cancer, since most of the regions are characterized by low coverage (The 1000 Genomes Project Consortium, 2015), and only blood rather than tissue samples are available. Nonetheless, this data set can help to address one of the major problems related to human evolutionary biology, that is, explaining how pigmentation variation evolved in different populations. Genes involved in melanogenesis are one of the major targets of natural selection, as verified by the correlation between pigmentation diversity and variation in ultraviolet (UV) incidence. Indeed, phenotypes distribution is clearly associated with latitude. Some theories have addressed natural selection and sexual dimorphism as possible explanations for this diversity (Aoki, 2002; Jablonski and Chaplin, 2014a). The most accepted evolutionary explanation concerning this pigmentation diversity refers to protection against UV radiation, particularly in skin areas that are highly exposed to sun. One of the differences between human beings and other primates, as well as many mammals, is that humans have lost most of the body hair. This fur loss should constitute an evolutionary advantage, such as the capacity to sweat more efficiently, which would help to regulate body temperature. However, the absence of this protective layer might have created the need for another type of protection against ultraviolet (UV) radiation (Rees, 2003). Dark skin pigmentation contains high levels of melanin, which can protect humans against UV damage (Jablonski and Chaplin, 2014b).

Alleles from many SNPs in the MC1R coding sequence (Arg151Cys, Arg160Trp, and Asp294His) have already been associated with red hair, fair skin, and freckling in Europeans (Harding *et al.*, 2000; Sturm, 2006). Other studies have also identified several *MC1R* coding variants related to melanoma susceptibility (Ghiorzo *et al.*, 2012; Pellegrini *et al.*, 2012). Understanding the expression and activity regulation of this receptor is essential to comprehend how it affects melanogenesis and the risk of developing melanoma (Swope *et al.*, 2012). Because natural selection shapes the global genetic diversity of regions with functional impact on the expression of genes and their products, understanding worldwide *MC1R* genetic diversity patterns is a first step to understand its expression and activity regulation.

Considering the limitations exposed here, we have employed a locally developed pipeline to obtain genotype and haplotype data from the raw sequencing data provided by the 1000 Genomes FTP site. We have also compared these data to Phase 3 VCF, which consists in the final release of a Variant Call File composed of about 80 million variant sites spread across the human genome, aiming to evaluate the quality of Phase 3 VCF and eventually discover any polymorphic sites that VCF may have overlooked.

## Subjects and Methods

This study used two different datasets maintained by the 1000 Genomes Project Consortium (Sudmant *et al.*, 2015; The 1000 Genomes Project Consortium, 2015).

The first dataset consisted of a VCF file obtained at the official website (http://browser.1000genomes.org/) from Phase 3 Release, which included 2,504 samples from 26 populations. The *MC1R* regions evaluated here (chr16:89981286-89987385) encompassed 1 kb from its 5’ Upstream Regulatory Region (5’URR), as well as its single exon composed of a 5’ Untranslated Region (5’UTR), a Coding Sequence (CDS), and a 3’ Untranslated Region (3’UTR).

The second dataset consisted of SAM files directly obtained from the 1000 Genomes server (ftp://ftp-trace.ncbi.nih.gov/1000genomes/ftp). Our group had previously validated the methodology used in this article (Castelli *et al.*, 2014). First, by using Samtools (Li *et al.*, 2009) subroutine view, we downloaded slices of the SAM (sequence alignment/map) files containing the 1000 Genomes data for the *MC1R* gene region mentioned above. We performed the download for all the 2,537 samples available in Phase 3 and included data from both low-coverage whole-genome and high-coverage exome sequencing when available. This process generated up to two SAM files per individual. We merged these two files into a single file and converted the resulting file into a BAM (binary alignment/map) file. We then converted each BAM file into a Fastq format file, which retrieved all reads previously mapped to the *MC1R* region, by means of Bamtools (https://github.com/pezmaster31/bamtools/) and Perl scripts (locally developed). Additional tools filtered out duplicated reads and classified the reads as paired or unpaired.

We then re-mapped both paired and unpaired Fastq files to a masked chromosome 16 (hg19), in which only the *MC1R* region was available; the rest of the chromosome was masked with “N” to preserve nucleotide positions regarding hg19. Picard-tools (http://broadinstitute.github.io/picard/) helped to join the BAM files resulting from the re-mapped reads, from both paired-end and unpaired sequences. The Bamtools software aided removal of reads mapped with low mapping quality (MQ) scores (MQ < 40). We used the GATK routines UnifiedGenotyper and HaplotypeCaller independently to infer genotypes and generate VCF (variant call format) files.

Given the low coverage nature of the 1000 Genomes data, some genotype callings are rather uncertain, mainly in situations in which a homozygous genotype is inferred when that position presents low depth coverage. In addition, given the polymorphic nature of *MC1R,* some level of mismapped reads is expected and might bias genotype inference. To circumvent this amplification bias issue, we treated both VCF files generated by UnifiedGenotyper and HaplotypeCaller with VCFx (http://www.castelli-lab.net/apps/apps_vcfx.php) a locally developed Perl script. This script uses the number of different reads detected for each allele at a given position (provided by both the GATK routines mentioned above upon generation of the VCF files) and applied the rules described below.

- Homozygosity was only inferred when a minimal coverage of seven reads was achieved; otherwise, a missing allele was introduced in this genotype. According to a binomial distribution and to different sets of simulations performed (data not shown), this procedure ensures (p > 0.99) that a homozygous genotype is called because of lack of variance at that position and not because the second allele was not sampled.
- Genotypes, in which one allele is extremely underrepresented (proportion of reads under 5%), are considered homozygous for the most represented allele. This procedure minimizes the influence of mismapped reads and the high level of sequencing errors that characterizes NGS data. Such correction is applied only in situations characterized by high depth of coverage (20 or more reads available for the evaluated position).
- For genotypes in which one allele is mildly underrepresented (with a proportion of reads between 5 and 20%), a missing allele is introduced representing this underrepresented allele. This procedure is particularly helpful in situations characterized by low depth of coverage (less than 20 reads available for the evaluated position), in which a single read may indicate the existence of an alternative allele. Such a read may be a mismapped read (false positive variant) or may represent a true unbalanced heterozygous genotype (true positive variant). Therefore, the definitive status of this kind of genotype (homozygous or heterozygous) was inferred during a final imputation step.
- Genotypes in which the proportion of reads for the less represented allele is higher than 20% are considered heterozygous. This procedure ensures that only high-quality heterozygous genotypes are passed forward to the imputation procedure.

It should be emphasized that the VCFx strategy is aimed to counteract amplification bias (Rehm *et al.*, 2013) and could be used in any NGS-derived VCF file. However, the rules described above would compromise the identification of somatic mutations or mosaicism, since these events would probably lead to unbalanced availability of reads incorporating alternative alleles, resembling PCR amplification bias

Using the VCFtools package (Danecek *et al.*, 2011), we removed SNVs that were no longer variable or that were represented just once in the dataset (*i.e.*, singletons). The missing alleles were imputed and *MC1R* haplotypes were inferred by using fastPHASE (Scheet and Stephens, 2006). A subset of the differences in genotype calling between the two approaches (UnifiedGenotyper and HaplotypeCaller) were visually inspected by checking the BAM files alignment by using the Integrative Genomics Viewer (IGV) 2.3 software (Robinson *et al.*, 2011).

### Statistical Analysis

The phased VCF file was converted into complete *MC1R* sequences using the hg19 reference sequence as a draft and replacing the correct nucleotide in each position, two sequences per samples, by applying VCFx function fasta (http://www.castelli-lab.net/apps/apps_vcfx.php).

Observed (*H*_*O*_) and expected (*H*_*S*_) heterozygosity values, as well as the adherences of genotypic proportions to expectations under Hardy-Weinberg equilibrium were estimated by the ARLEQUIN version 3.5 program (Excoffier and Lischer, 2010). The *MC1R* sequence variation was assessed using θ_*W*_, an estimate of the expected per-site heterozygosity, and π (nucleotide diversity), which is the average number of nucleotide differences per site between two sequences (Nei and Kumar, 2000).

Departure from selective neutrality was tested by four different methods. The first one was the Ewens-Watterson test (Ewens, 1972; Watterson, 1978; Slatkin, 1994). This test, based on Ewens’ sampling theory and infinite allele model, compares the observed homozygosity under Hardy-Weinberg proportions with the expected homozygosity computed by simulation under the hypothesis of neutrality/equilibrium expectations, for the same sample size and number of alleles. This permits to test alternative hypotheses of either directional (observed homozygosity greater than expected homozygosity) or balancing selection (observed homozygosity lower than expected homozygosity). The second method was the Tajima’s *D* test (Tajima, 1989), which examines the relationship between the number of segregating sites and nucleotide diversity, by comparing the sequence diversity statistics θ_*W*_ and π. Under the standard neutral model, the expectations of θ_*W*_ and π are equal, and therefore the expected value of Tajima’s *D* is zero under neutrality. A positive Tajima’s *D* value evidences heterozygous advantage and a negative value points to selection of one specific allele over alternate alleles (Nei and Kumar, 2000). Like Tajima’s *D*, the third test, Fu’s *F*_*S*_ test (Fu, 1997) is based on the infinite-site model without recombination. It evaluates the probability of observing a random neutral sample with a number of alleles similar or smaller than the observed value given the observed number of pairwise differences, taken as an estimator of θ. Considered less conservative than Tajima’s D, Fu’s *F*_*S*_ is more sensitive to the presence of singletons. The significance of the Tajima’s *D* and Fu’s *F*_*S*_ statistics were tested by generating 99,999 random samples under the hypothesis of selective neutrality and population equilibrium, using a coalescent simulation algorithm. These three neutrality tests were carried out using the ARLEQUIN version 3.5 program (Excoffier and Lischer, 2010). The fourth method consisted of the synonymous and non-synonymous nucleotide substitution test, which evaluates the relative abundance of synonymous substitutions (that do not result in amino acid change) and non-synonymous substitutions (that result in amino acid change) which occurred in the gene sequences. For data sets containing more than two sequences, this is done by first estimating the average number of synonymous substitutions per synonymous site (*dS*) and the number of non-synonymous substitutions per non-synonymous site (*dN*), and their variances. Then, the null hypothesis of neutrality (*dN = dS*) can be evaluated against the alternative hypothesis of either positive or purifying selection. This test was carried out using the Nei-Gojobori method (Nei and Gojobori, 1986) implemented in MEGA version 7.0.21 (Kumar *et al.*, 2016).

## Results

After the steps that converted the raw data of the 1000 Genomes Project to the phased haplotypes of *MC1R* for all individuals, the process that used the GATK routine UnifiedGenotyper resulted in complete data for 201 *loci* for 2,537 individuals, which represented 596 distinct haplotypes. Of these 201 *loci*, 24, 54, 89 and 34 belonged to 5’URR (Upstream Regulatory Region), 5’UTR (Untranslated Region), CDS (Coding Sequence), and 3’UTR, respectively. As the data obtained directly from the 1000 Genomes Project VCF had 178 *loci* (174 SNVs and 4 InDels) that were common to the same 2,504 individuals, we removed the 23 *loci* (11 singletons) and 33 individuals that were incompatible between the two datasets, which allowed equivalence in comparisons.

After this initial filtering, we compared genotypes for each *locus* among individuals to verify whether incompatibilities existed. An initial analysis considering the total number of *loci* (178) and individuals (2,504) revealed that 445,712 possible genotype comparisons could be performed between the results of the UnifiedGenotyper analysis and the 1000 Genomes browser data. From this total, there were only 585 incompatibilities in terms of genotype calling (0.1313% of the total).

**Figure 1:**
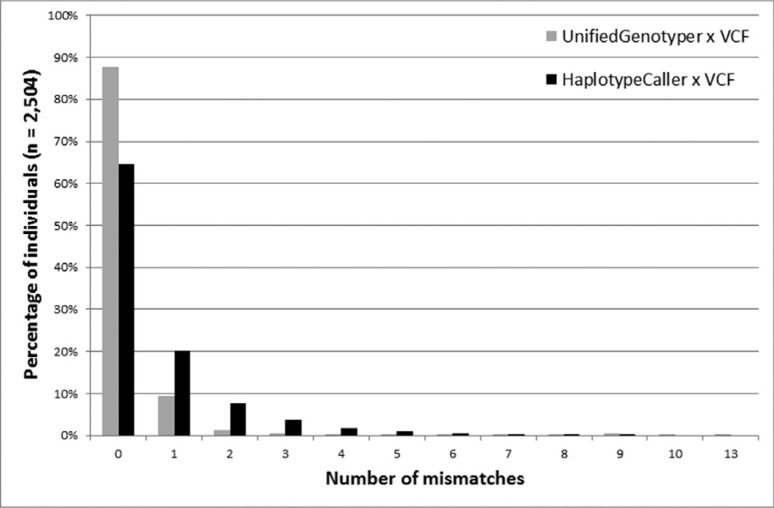
Percentage of mismatches observed for data generated by our pipelines (UnifiedGenotyper in gray and HaplotypeCaller in black) as compared to data obtained directly from the VCF concerning 2,504 individuals analyzed by the 1000 Genomes Project.

**Figure 2:**
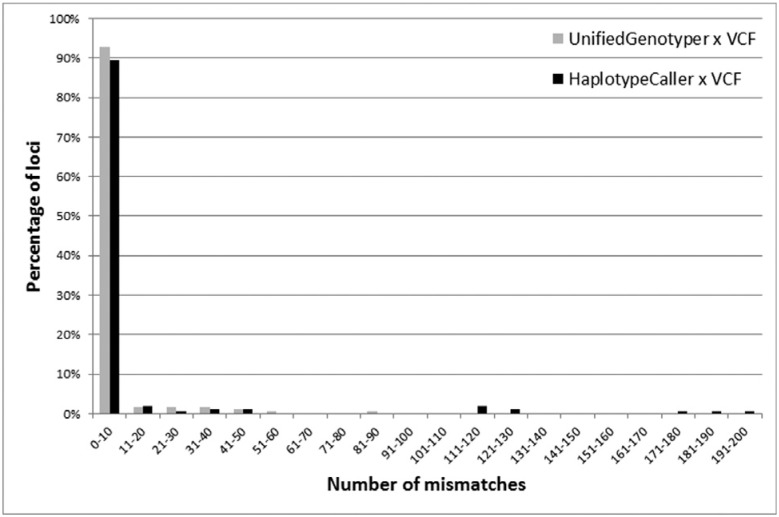
Percentage of mismatches observed for data generated by our pipelines (UnifiedGenotyper in gray and HaplotypeCaller in black) as compared to data obtained directly from the VCF concerning 178 (UnifiedGenotyper) or 150 (HaplotypeCaller) *loci* analyzed by the 1000 Genomes Project.

For all the analyzed loci, the two datasets provided identical genotype data for 87.58% of the sample (2,193 individuals). The remaining 311 subjects had at least one *locus* with an incompatible genotype, and this error rate reached a maximum of 13 incompatible *loci* in a single individual (Figure 1). According to the two approaches, individual analysis of each *locus* showed that 102 of the *loci* were compatible for all the individuals. Among the 76 discordant *loci*, some condensed most of the discrepancies: 13 of these *loci* accounted for almost 80% of all the mismatches throughout the study, and one of them (rs885479) led to 87 mismatches (14.87% of total) (Figure 2).

Analysis of the allele frequencies for each evaluated *locus* demonstrated that the two approaches did not differ significantly despite the mentioned mismatches. In the end, even *locus* rs885479, which presented the largest amount of mismatches, provided an irrelevant difference of 1.22% for the reference allele frequency determined by the VCF and by the data processed via our scripts.

Based on these results, we checked the BAM files alignment by using IGV software to explore the mismatches between the two approaches manually. From the original 585 genotype differences, we evaluated 40 random mismatches for *locus* rs885479 and 60 mismatches randomly chosen among the remaining *loci* (Table 1). From the 40 mismatches evaluated for *locus* rs885479, 39 had seven reads or less and, therefore, resulted in the introduction of a missing allele by the script, which made data comparison uninformative. The other mismatch apparently occurred due to some genotype calling error in our pipeline (it was wrongly considered homozygous). The pipeline introduced missing alleles in 33 of the 60 random mismatches either because the read number was low or because the alternative allele was underrepresented, which made data comparison uninformative. Other 22 mismatches were due to errors made by our pipeline (the pipeline should have regarded six and 12 cases as homozygous and heterozygous, respectively; the four remaining cases were considered homozygous for the reference allele while they should have been considered homozygous for the alternative allele), and in five cases the VCF made erroneous genotype calling.

**Table 1:** Comparison between the 1000 Genomes Project Phase 3 VCF with data processed by the pipelines that include either UnifiedGenotyper or HaplotypeCaller. For this purpose, 40 callings were retrieved from the *locus* that presented the higher levels of inconsistencies (rs885479 for UnifiedGenotyper and chr16:89985177 for HaplotypeCaller) and 60 were randomly chosen among the remaining *loci*.

	UnifiedGenotyper approach		HaplotypeCaller approach	
Mismatches	*Locus* rs885479	Random sites	chr16:89985177	Random sites
**Missing Alleles**	39	33	38	56
**VCF error**	0	5	0	1
**Pipeline error**	1	22	2	3

Surprisingly, the 178 *loci* in the 2,504 individuals, which resulted in 207 distinct haplotypes for the VCF data, ended up resulting in 491 different haplotypes in the data generated by our UnifiedGenotyper pipeline. Not all 207 haplotypes obtained from VCF were among those obtained by the scripts. From the total, 52 haplotypes were not among the 490 haplotypes inferred by our pipeline. Comparing the haplotype pairs obtained for each individual, of the 5,008 generated haplotypes, 2,442 haplotype comparisons were identical and 2,566 included some inconsistencies. This result became even more astonishing when considering that the two datasets gave identical genotype data for all the analyzed *loci* for 87.58% of the sample (2,193 individuals), but only 1,147 individuals (52.3%) had identical haplotype reconstructions.

The GATK routine HaplotypeCaller afforded data for 163 *loci* in 2,537 individuals. Because some incompatibilities also existed between this dataset and the 1000 Genomes VCF, we removed 33 individuals and 28 *loci* to allow equivalence in comparisons. This second approach resulted in 375,600 possible genotype comparisons (150 *loci* x 2,504 individuals). This comparison resulted in 1,637 genotype calling incompatibilities (0.4358% of the total).

For all the analyzed *loci*, the two datasets revealed identical genotype data for 64.54% of the sample (1,616 individuals). At least one incompatible *locus* existed in the remaining 811 individuals, and this error rate reached a maximum of eight incompatible *loci* in a single individual (Figure 1). Considering each *locus* individually, the two approaches revealed compatibility for 54 of the *loci* for all the individuals. Some of the 103 discordant *loci* condensed most of the discrepancies: 13 *loci* accounted for almost 85% of all mismatches throughout the study, and one *locus* (position chr16:89985177) led to 184 mismatches (11.93% of the total) (Figure 2).

Considering the allele frequencies for each *locus*, we again observed no significant differences in this second analysis despite the mismatches (maximum of 0.48% per *locus*). Even for *locus* chr16:89985177, which comprised most mismatches, VCF and data processed by HaplotypeCaller differed by only 0.02% in allele frequencies.

We also checked the BAM files alignment manually to better understand these mismatches. As in the case of the first comparison (UnifiedGenotyper *vs* VCF), we evaluated 40 random mismatches for *locus* chr16:89985177 and 60 random mismatches among the remaining *loci* (Table 1). Of the 40 mismatches evaluated for *locus* chr16:89985177, only one mismatch had more than seven and less than 20 reads, with an underrepresented alternate allele (ratio between 5-20%), which resulted in the introduction of a missing allele. The HaplotypeCaller pipeline considered another 37 mismatches as missing alleles, which made data comparison uninformative. Two mismatches probably occurred due to some genotype calling error in our pipeline (both should have been considered as heterozygous), but VCF did not result in any wrong genotype calling. Evaluation of 60 random mismatches showed that 56 of them had low coverage (less than seven reads): the pipeline considered 54 of these mismatches as missing alleles and wrongly considered the other two as homozygous. As for the remaining four mismatches, two were considered as missing alleles, one was wrongly mapped as heterozygous by the pipeline, and one was considered as heterozygous by the VCF.

Comparison between the 1000 Genomes browser and the HaplotypeCaller approach (150 *loci* in 2,504 individuals) showed 181 unique haplotypes for the VCF data and a surprisingly higher number of 727 distinct haplotypes for our HaplotypeCaller pipeline. Forty-five of the haplotypes inferred by 1000 Genomes were not present in our pipeline results. Comparison of the individual haplotype pairs revealed a similar number of incompatibilities (2,444 identical comparisons out of 5,008).

Finally, in order to evaluate the population genetics applicability of the 1000 Genomes dataset, characterized by low coverage sequencing, basic population genetics parameters and four different neutrality tests were applied to all four population groups composed of autochthonous populations (Tables 2 and 3).

**Table 2:** Observed (*H*_*O*_) and expected (*H*_*S*_) heterozygosities, Hardy-Weinberg Equilibrium (*HWE*) probability values and Ewens-Watterson neutrality test results regarding the promoter and coding regions of *MC1R* in four population groups composed of autochthonous populations from the 1000 Genomes project. Significant *p*-values are marked in boldface.

					Ewens-Watterson neutrality test		
	*2n*	*H*_*O*_	*H*_*S*_	HWE *p*-value	*F*_*observed*_	*F*_*expected*_	*p*-value^a^
**Promoter region**							
AFR	1008	0.881	0.883	0.537	**0.118**	**0.049**	**1.000**
EAS	1008	0.639	0.625	0.617	**0.376**	**0.104**	**1.000**
EUR	1006	0.692	0.689	0.499	**0.312**	**0.123**	**1.000**
SAS	978	0.847	0.839	0.613	**0.162**	**0.062**	**1.000**
**Coding region**							
AFR	1008	0.669	0.666	0.494	**0.334**	**0.130**	**1.000**
EAS	1008	0.587	0.565	0.546	**0.436**	**0.169**	**1.000**
EUR	1006	0.638	0.668	0.584	**0.333**	**0.200**	**0.959**
SAS	978	0.481	0.491	0.425	**0.510**	**0.184**	**1.000**

a A *p*-value was computed by the comparison of the estimated statistic to a distribution of estimates computed for 99,999 random samples of the same number of alleles and sample size as the observed data, and represents the proportion of samples having a probability smaller or equal to the observed sample. Due to the nature of the test, large *p*-values (*i.e.*, p > 0.95) are still significant.

**Table 3:** Summary of sequence variation in the promoter and coding regions of the *MC1R* gene in four population groups composed of autochthonous populations from the 1000 Genomes project and results of three neutrality tests based on sequence data: Tajima’s *D* test, Fu’s *F*_*S*_ test, and synonymous and non-synonymous nucleotide substitution test (*dN - dS*) of positive and purifying selection for analysis averaging *MC1R* coding haplotypes. Significant *p*-values are marked in boldface.

							Tajima’s *D* test		Fu’s *F*_*S*_ test		*dN* / *dS* nucleotide substitution test		
	*2n* a	Number of nucleotide sites	*K* b	*S* c	θ_*W*_ ± SD	π ± SD (%)^d^	*D*	*p*-value^e^	*F*_*S*_	*p*-value^f^	Number of codons	H_A_ = Positive Selection	H_A_ = Purifying Selection
												(*dN* > *dS*)	(*dN* < *dS*)
**Promoter region**													
AFR	1008	3001	78	55	6.140 ± 1.367	0.224 ± 0.117	-0.991	0.152	**-24.074**	**0.003**	-	-	-
EAS	1008	3001	42	45	4.805 ± 1.131	0.188 ± 0.100	-1.024	0.136	-4.521	0.210	-	-	-
EUR	1006	3001	36	42	4.272 ± 1.036	0.164 ± 0.089	-0.808	0.227	-3.360	0.291	-	-	-
SAS	978	3001	64	56	6.298 ± 1.399	0.213 ± 0.112	-1.030	0.157	**-17.287**	**0.017**	-	-	-
**Coding region**													
AFR	1008	954	33	31	4.138 ± 1.012	0.093 ± 0.072	**-1.964**	**0.000**	**-27.698**	**0.000**	316	-1,426; p = 1,000	1,453; p = 0,074
EAS	1008	954	26	20	2.669 ± 0.741	0.150 ± 0.102	-1.078	0.128	**-13.226**	**0.006**	317	-0,142; p = 1,000	0,141; p = 0,444
EUR	1006	954	22	22	2.937 ± 0.792	0.103 ± 0.077	**-1.578**	**0.021**	**-13.894**	**0.001**	317	0,444; p = 0,329	-0,439; p = 1,000
SAS	978	954	24	23	3.082 ± 0.821	0.062 ± 0.055	**-1.939**	**0.001**	**-26.733**	**0.000**	317	-1,034; p = 1,000	1,055; p = 0,147

a *2n*: number of chromosomes analyzed

b Number of different haplotypes

c Number of segregating sites

d Average nucleotide diversity and its standard deviation

e A *p*-value was computed by the comparison of the estimated statistic to a distribution of estimates computed for 99,999 random samples of the same sample size and level of polymorphism as the observed data, and represents the proportion of the simulated *D* statistics less or equal to the observed value.

f A *p*-value was computed by the comparison of the estimated statistic to a distribution of estimates computed for 99,999 random samples of the same sample size and level of polymorphism as the observed data, and represents the proportion of the simulated *F*_*S*_ statistics less or equal to the observed value. The 2% percentile of the distribution corresponded to the 5% cutoff value. Therefore, a *F*_*S*_ statistic should be considered as significant at the 5% level, if its *p*-value is below 0.02, and not below 0.05.

## Discussion

It is well established that low-coverage next-generation sequencing assays are deeply affected by PCR amplification and sequencing biases. According to Rehm *et al.* (2013), low coverage associated with amplification bias increases the risks of missing variants and assigning incorrect genotypes. It also decreases the ability to effectively filter out sequencing artifacts, leading to false-positives. The three platforms used by the 1000 Genomes Consortium (454, Illumina and SOLiD) display systematic biases and unevenness (Aird *et al.*, 2011). According to Meynert *et al.* (2014), data generated by the 1000 Genomes Project present a bad performance related to detection of heterozygous SNPs, which could be due to short read lengths or high sequencing error rates, requiring a coverage of at least 13X to reach 95% sensitivity. Therefore, many called homozygotes are actual heterozygotes, as well as amplification and sequencing artifacts may lead to false-positives (Rehm *et al.*, 2013). One way to deal with this issue is by introducing protocol modifications (Aird *et al.*, 2011). Since this is not a possibility when dealing with data as the already obtained by the 1000 Genomes Project, designing methods to interrogate and enhance genotype calling accuracy is of paramount importance.

Most of the evaluated mismatches found for the datasets originated from the high level of missing alleles (72% for UnifiedGenotyper and 94% for HaplotypeCaller, Table 1), which resulted mainly from the low coverage (below seven or 20 reads) of the analyzed polymorphic sites. This led us to include imputation steps in our pipeline. Hence, the high inconsistency ratio between the VCF file and the genotypes obtained by both pipelines probably reflected the differences concerning the imputation methods used in each approach and cannot be straightforwardly considered as error. At the moment, empirically stating which imputation method is more prone to error is not possible in such extremely low-coverage situations.

Genotype imputation can be extremely useful by allowing missing data from low-density chips to be filled, which should reduce costs (Jimenez-Montero *et al.*, 2013), merge datasets with non-overlapping genotypes generated by different platforms (Khankhanian *et al.*, 2015), or even predict complex traits (Felipe *et al.*, 2014). The accuracy of imputed genotypes, in cases where an appropriate reference population is used, resembles the accuracy obtained from high-density SNP panels (Weigel *et al.*, 2010). However, imputation benefits rely deeply on the accuracy of the imputation procedure, which is directly related to the population structure of the samples used and to the composition of the evaluated target gene (Felipe *et al.*, 2014).

Although low coverage is a recurrent problem regarding NGS of whole genomes, another important issue is the read mapping bias. This is because highly polymorphic regions will generate reads consisting of many alternate alleles. Some of these greatly diverse reads will not align to a single position in the reference genome and, hence, will be discarded during analysis. Therefore, it would be interesting to have multiple haplotypes from a given target as reference for several alignment steps in order to improve genotype calling for these polymorphic regions (Brandt *et al.*, 2015).

Most studies that use genotype imputation for whole-genome sequencing data regard only linear prediction models such as ridge regression, Bayesian LASSO (least absolute selection and shrinkage operator), or GBLUP (genomic best linear unbiased prediction) approaches. These approaches are more suitable for additive genetic models but do not detect so accurately non-additive effects, such as dominance and epistasis (Weigel *et al.*, 2010; Felipe *et al.*, 2014).

However, even different imputation models can present bias in specific cases. Examples of such cases are high heterogeneity that culminates in underestimation of derived alleles, presence of the alternative allele in small genetic windows that are not spanned by enough SNPs to make imputation effective, and rare frequency of the alternative allele. Although the imputation process can lead to more powerful datasets and new association in case-control or population studies, the inherent bias can lead to data deficiency or type I error, which could counteract the results from these datasets by missing or underestimating genotype associations (Khankhanian *et al.*, 2015).

Therefore, it is essential to evaluate the genetic structure of the data accurately, in order to choose the best fitting and most reliable imputation method for the investigated genes or genomic regions. It is crucial not only to choose the most suitable imputation method for the presented data, but also to use a validation regression method to examine the results, search for artifacts, and ensure method validity (Hoffmann *et al.*, 2015), or use other established and validated methodologies, such as Sanger sequencing.

Finally, compared to the 1000 Genomes browser VCF data, UnifiedGenotyper (0.1313%) and HaplotypeCaller (0.4358%) led to a very small number of inconsistencies. Although HaplotypeCaller afforded a higher rate of inconsistencies, 94% of the inconsistencies were related with interrogation of low coverage genotypes rather than with VCF (1%) or pipeline (5%) errors used for genotype calling. In turn, UnifiedGenotyper was associated with higher levels of VCF (5%) or pipeline (23%) errors, such as detection of homozygotes as heterozygotes and vice versa. Therefore, the fraction of inconsistencies that represented actual pipeline errors was estimated as 0.0302% and 0.0218% for HaplotypeCaller and Unifiedgenotyper, respectively. The initially apparent worse performance of HaplotypeCaller must indeed have been due to the fact it is a conservative approach and therefore required higher-quality raw data (*i.e.*, characterized by higher coverage, base quality, and mapping quality) to be reliable. Although the inaccuracies of the pipelines deserve further investigation, the most plausible cause of such discrepancies could be related to the fact that part of the reads aligned to a given position, and when confirmed by visual inspection with IGV did not present the quality requirements necessary for consideration by GATK routines for calling. This could also underlie the smaller number of variation sites identified by HaplotypeCaller (163) as compared to UnifiedGenotyper (201) and to Phase 3 VCF (178). A total of 150 variation sites were identified by the three methods. Overall, HaplotypeCaller and UnifiedGenotyper identified 13 and 23 variants, respectively, that were previously overlooked by the Consortium; seven of them were identified by both algorithms, which increases the likelihood of being real. Although these variants still demand confirmation, their impact on most of the population genetic analyses is negligible since most of them are singletons.

Data from next-generation sequencing should be treated carefully if one wishes to obtain the most accurate information. The present comparison evidenced a very low incompatibility rate in terms of *MC1R* genotype calling (below 0.5%), and our preliminary analysis of the 1000 Genomes dataset has disclosed solid signatures of purifying selection on African and positive selection on European populations in both the promoter and the coding region of *MC1R*. According to UV incidence data, AFR and SAS populations are related with places of higher UV incidence and higher melanin content (Jablonski and Chaplin 2000), when compared to EUR and EAS, which may suggest different selective pressures on pigmentation genes. Reports of natural selection shaping *MC1R* diversity already proposed positive selection in Eurasia (Savage *et al.*, 2008; Hider *et al.*, 2013). In the present dataset, the Ewens-Watterson test revealed a significant deficiency of heterozigosity in the promoter and coding regions of all four population groups composed of autochthonous populations (Table 2), irrespective of the different demographic histories that characterize each population, which is consistent with directional selection that may be either positive or purifying selection.

All Tajima’s D and Fu’s *F*_*S*_ values for the *MC1R* coding region were more negative than the respective values for the promoter region in all population groups (Table 3), indicating an excess of rare variants, which is consistent with either positive or purifying selection, particularly on the coding region. AFR and SAS presented lower (more negative) values than observed for EUR and EAS in both tests. Although both positive and purifying selection may result in similar signatures, the latter results in even reduced levels of variability (Nielsen, 2005), which was observed in AFR and SAS when compared with EUR and EAS when nucleotide diversity of coding region is considered (Table 3). A good method to tell purifying apart from positive selection, i.e, identify the directionality of selection, is to apply the synonymous and non-synonymous nucleotide substitution test (*dN - dS*). However, as purifying selection will tend to dominate in evolution, this becomes a very conservative tool and the amount of positive selection needed to to be detectable is enormous (Nielsen, 2005). Although far from reaching statistical significance, the test results suggests that positive selection should be more appropriate to explain the significant findings of directional selection revealed by the other three tests in EUR.

The present *MC1R* study reveals that the 1000 Genomes dataset is adequate to perform population genetic studies. The fact that it was not possible to retrieve any multiallelic marker for the *MC1R* region calls for conduction of similar studies including multiallelic markers. Nevertheless, the present comparison suggests that either the VCF file or one of the presently described pipelines could be used to obtain reliable and accurate genotype calling during studies of individual genes or delimited chromosome regions from 1000 Genomes Phase 3 dataset. A negative issue that needs further examination is the significant disagreement found for the haplotype inference procedure, which may have resulted from underestimation of haplotypes in the VCF file or from overestimation by fastPHASE. Although low coverage can be a great problem regarding this kind of analysis, imputation methods can be useful to fill missing data gaps, as long as an accurate reference panel is chosen. Higher coverage could also improve the precision of genotype calling, but the development of new methods to identify and correct for bias could be the only answer in cases where only low-coverage data is available.
